# Notes and Comments

**Published:** 1898-07

**Authors:** 


### Notes and Comments.[1]

Do the Nerves grow Old?—Commenting on the common causes of nervous disorders, Professor W. H. Thomson says, "The message of modern science about the nervous system is more hopeful than ever. It tells us that the nervous system has a greater store of reserve vitality than all the other bodily systems put together. It is the only texture that is found not to have lost weight after death by starvation, as well as after death by any cause. It is the last to grow old; and, as to the mind, it need not grow old at all, provided it be steadily applied with that mighty spiritual element in us which we call interest. Even the muscular system can be wonderfully sustained by interest; for should a man attempt the same muscular work on a treadmill which he lightly endures along the mountain brook after a trout he would faint dead away. But the mind will by interest grow steadily, even while bone and sinew are wasting through age."

---

Gold in Amalgam.—Dr. W. W. Coon, in a paper read before the Eighth District Dental Society, Buffalo, N. Y., says, "I have never found an amalgam alloy that was not improved in the using by the addition of gold. I have spoken of the matter occasionally to dentists, and in a few instances to manufacturers, and the usual reply has been founded on what has appeared in print,—viz., 'uncertain usefulness.'"

The doctor says that the uncertainty has long since vanished from his mind, as he quite often sees in the mouth of some old patient an amalgam filling that attracts attention because it is better than the others he has inserted in the same mouth, and, in looking it up in his records, finds that gold is the differentiating factor.

---

Removal of Teeth from Rubber Plates.—It sometimes falls to one's lot to remove teeth from an old rubber base. It is com-

[1] The assistant editor solicits contributions for this department,—new methods, new remedies and formulas, or any short practical note which may prove of value to the practitioner or student. Address 1338 Walnut Street, Philadelphia.

mon to proceed by holding the plate over a gas-jet until the teeth can be pried off, and in doing so operators seem to forget that the odors of $H_2S$, $S_2O$, and other gases have found their way to the remotest corner of the room, and possibly to adjoining premises. This might be avoided by boiling the plate for a few moments, when the rubber will be found yielding, and by grasping it with the pliers the rubber may be sprung from the teeth, and a few repetitions will complete matters.—*Ohio Dental Journal.*

